# VR Health Experience: A Virtual Space for Arts and Psychomotor Therapy

**DOI:** 10.3389/fpsyg.2021.704613

**Published:** 2021-09-14

**Authors:** Suzanne Haeyen, Nathalie Jans, Marleen Glas, Joep Kolijn

**Affiliations:** ^1^GGNet Centre of Mental Health, Department Scelta, Apeldoorn, Netherlands; ^2^Department Special Research Group Arts & Psychomotor Therapies in Personality Disorders, HAN University of Applied Sciences, Nijmegen, Netherlands

**Keywords:** telepresent, art therapies, VR Health Experience, VR therapy, psychomotor therapy, online, mental health, virtual reality

## Abstract

**Introduction:** Mental health and well-being are under pressure because of the corona pandemic. Arts and psychomotor therapists said that they had almost no experience with working online, but despite the fact that they felt incapable, they were positive towards it.

**Method:** This qualitative action research was aimed at how arts and psychomotor therapists can become more skilled in offering online arts and psychomotor therapy and how they can methodically enlist the VR Health Experience (a virtual arts and psychomotor therapy space) in therapy. It is envisaged that the arts and psychomotor therapist could be telepresent in order to offer arts and psychomotor therapy remotely. In online training that made use of the Lean Startup Method, participants (*n* = 5) integrated their working knowledge with the VR Health Experience. The interventions were immediately tested by the participants and in practice. Participants were interviewed retrospectively and their experiences were thematically analysed.

**Results:** The VR Health Experience came forward as an innovative addition to the usual arts and psychomotor therapy. Often, clients were encouraged to play and experiment, and the VR world offered several options. The VR Health Experience lowered the threshold, expanded the training areas and held a great attraction for play.

**Discussion:** This project offered an innovative quality boost for arts and psychomotor therapy. Arts and psychomotor therapy have proven to be possible remotely via telepresence of the therapist. The therapist is present together with the client in the VR Health Experience, the virtual arts and psychomotor therapy space, where connectedness can be felt and new experiences can be gained. VR also offers further possibilities in arts and psychomotor therapy.

## Introduction

The COVID-19 measures have a huge influence on society. This applies all the more to vulnerable groups, such as people with psychological or psychiatric diagnoses or symptoms. Their health and welfare is under pressure. On account of these same measures, treatments of various target groups in mental health care are delayed or temporarily put on hold. This also applies to the arts and psychomotor therapeutic treatments, such as visual art, dance, drama, music, play, and psychomotor therapy, for a variety of target groups in mental health care. Arts and psychomotor therapies have an experiential, action-oriented, creative quality. These therapies methodically and purposefully use various forms of work, materials, instruments, and attributes (Malchiodi, 2012; American Art Therapy Association (AATA), 2021; British Association of Art Therapists (BAAT), 2021). Psychomotor therapy^1^, an internationally less known profession or term, uses interventions that focus on movement behaviours and/or body experience (FVB, 2021; Nederlandse Vereniging voor Psychomotorische Therapie (NVPMT), 2021). Just as the arts therapies, these interventions are, mainly based on experience knowledge and offer the client the opportunity to acquire new skills, gain meaningful and insightful experiences or modify intrapsychic, interpersonal or systemic conflicts. Arts and psychomotor therapies have a long history of clinical success with various populations and there is a limited but growing and promising increase of scientific support for the intervention strategies and theoretical models involved (e.g., Czamanski-Cohen and Weihs, 2016; Kaimal et al., 2016, 2017; Haeyen, 2018; Haeyen et al., 2018; King and Parada, 2021).

The COVID-19 pandemic forced a sudden change in practice and a move into online delivery for many arts and psychomotor therapists, often with minimal guidance and little previous experience of remote delivery (Haeyen et al., 2020; Zubala and Hackett, 2020). At present, those working in mental health care give due regard to the social distance that is recommended by local authorities, and this will probably continue for some time. One-quarter of the respondents of a large survey (*N* = 840; 91% client, 8% family member, 1% partner) indicated that they now had partly online and partly face-to-face contact, and one in eight had online contact only (MIND, 2021). Depending on developments and the outbreak of virus variants, it is possible that a great many clients will need to be treated online. On account of a chronic disorder, some clients must wait with face-to-face contact until everyone has been vaccinated. The consequences are huge.

In addition to existing complaints, clients had extra emotional problems as well on account of the corona crisis. An online survey by Trimbos Institute among the general population (3000 people) showed that 1 in 3 respondents had more anxiety, depressive complaints and sleep problems during the corona crisis. Among the participants who experienced more stress on account of the corona pandemic, 1 in 10 had suicidal thoughts. Nearly half of these respondents were in need of help (Trimbos Institute, 2020).

Covid-19 can have both physical and mental consequences. The infection itself can be physically devastating, but the psychological toll of experiences can be high as well, such as loss of family or friends to the illness, loss of a job, or loneliness due to isolation in complying with the corona measures (staying at home, keeping distance, etc.), according to Harvard T. H. Chan School of Public Health (2021). After some time - now one year later - the impact of corona and the corona measures have significantly increased for people with mental distress. There is an increase in symptoms such as sombreness, depression and anxiety, growing tension and stress, loneliness and difficulty sleeping (MIND, 2021; Trimbos Institute, 2021). Three out of four people experience negative consequences, and those between 20 and 35 prove to have the greatest mental distress (MIND, 2021). Because of this, and due to their loss of work, they wind up in social isolation. Gaining perspective and acquiring more opportunities for social contact prove to be important to bring them through the ongoing crisis in good condition (Trimbos Institute, 2021). Considering the great impact of corona and the corona measures on mental welfare, continuity of care and treatment is essential (MIND, 2021).

Put briefly, providing online help is extremely urgent, since people must more often stay in quarantine. In this exceptional situation it is important to target all our efforts on people’s resilience to deal with the challenges of this day and age, in order to increase their well-being and welfare. Health is the capacity to adjust and control your own life in the light of the physical, emotional, and social challenges it brings (Huber et al., 2011). People with mental or psychiatric problems and issues often have difficulty with this resilience and often have trouble dealing with emotions.

For professionals, including arts and psychomotor therapists, it proved difficult to suddenly have to give online therapy, without any experience and without sufficient knowledge of online treatment. A survey (Haeyen, 2020) of the experience of 281 Dutch arts and psychomotor therapists in giving online therapy showed that people basically have “no experience whatsoever” (77.2%) or “almost no experience” (13.9%) with giving online art and psychomotor therapy. Even though people are “reasonably” to “highly positive” (35.9 and 10.7%), they feel “somewhat incompetent” to “highly incompetent” to offer it (26.7 and 4.6%). Such surveys were held internationally in the United Kingdom and the United States as well (Choudhry and Keane, 2020; Zubala and Hackett, 2020; 96 and 623 arts therapists, respectively). The respondents expressed concerns about safety of practice as well as about their own confidence in delivering therapy remotely. Increased clinical supervision, specialist training, and support from colleagues were appreciated and valued in the rapid transitioning to online practice. Arts and psychomotor therapists have stepped out of their comfort zones to get creative virtually, incorporating the art-making process into teletherapy sessions and connecting with their clients in new ways during this pandemic. The need for greater knowledge also came forward explicitly in the large number of participants (680) for a master class of online arts and psychomotor therapy, given at the beginning of the corona pandemic (Kolijn et al., 2020).

Arts and psychomotor therapies are perceived to be more difficult to carry out at a distance, as shown by the survey among therapists (*N* = 281, Haeyen, 2020), because they work experientially with various media (artistic, expressive, and action-focused, such as music, drama, working visually, body consciousness, movement, play, etc.). By working experientially, clients become more skilful and proficient; they develop more identity/self-confidence and conscious insight into thinking, feeling and acting on their own in certain contexts. The strength of arts therapy for the client lies not only in *knowing* what to do, but also in actually *acting*, literally carrying out and making use of the behavioural change. Offering therapy at a distance, on the basis of this characteristic aspect, is a challenge for arts and psychomotor therapists.

There are a number of reasons why giving online therapy did not immediately get off the ground at the beginning of the first lockdown, as came forward in the answers to the survey among the therapists (Haeyen, 2020). United Kingdom -based art therapists as well needed to adopt creative approaches to make a rapid shift to delivering therapy online, often with minimal guidance and little previous experience of remote delivery. The respondents (*N* = 96) expressed concerns about safety of practice and their own confidence in delivering therapy remotely (Zubala and Hackett, 2020). During the corona crisis it became clear that knowledge of it, and the means by which arts and psychomotor therapists can offer online arts and psychomotor therapy, are both very limited. The first factor was that arts and psychomotor therapists do not feel competent and confident (Haeyen, 2020; Zubala and Hackett, 2020). A second factor was that arts and psychomotor therapists have limited access to digital tools. An arts therapist who works in an institution does have access to a digital environment or an eHealth platform, but this platform is primarily set up for interventions aimed at focusing on talk therapy. The means are even more limited for independently established arts and psychomotor therapists. They use the conventional, and unfortunately not always completely private, video call options such as WhatsApp, Zoom, FaceTime, etc. A third factor is that the tools available do not connect well to arts therapy: tools are often not specifically equipped for the arts and psychomotor therapies. Working experientially as starting point in arts and psychomotor therapies demands extra attention as well as solutions. Finding the right telepresence tool to make it possible to work experientially at a distance is therefore the challenge. Extended reality such as Virtual Reality (VR) seems suited to action when no face-to-face contact is possible.

VR is in development and is more and more often enlisted in treating people with mental complaints, as appears from a number of publications and investigations. The capacity to simulate different realities and experiences has also prompted the use of VR in psychotherapy, where VR techniques have been implemented in the treatment of phobias, PTSD, and anxiety disorders (Rothbaum et al., 2002; Parsons and Rizzo, 2008; Beidel et al., 2017), depression (Falconer et al., 2016), schizophrenia, eating disorders (Gutiérrez-Maldonado et al., 2016), and pain management (Freeman et al., 2017; in Hacmun et al., 2018). For example, CleVR (2021), in co-creation with researchers and clinicians from practice, developed dynamic interactive virtual social worlds for effective behavioural change, known under the name of Virtual Reality Exposure Therapy (VRET; Lindner et al., 2017). CleVR developed a number of VR products that are used by several training courses (in particular, aggression regulation and psychopathology) for clients and professionals in mental health care and the forensic sector, including, for example, VRAPT (Virtual Reality Aggression Prevention Therapy). CleVR describes how VR-Cognitive Behavioural Therapy (VR-CBT) is presently applied successfully to treat various mental problems - for example, a psychotic disorder (including anxiety and paranoia), social anxiety disorder, panic disorder, depressive disorder, autism, and anxiety complaints in the context of a personality disorder (CleVR, 2021). In the virtual world developed by CleVR, both one on one conversations between virtual characters and group conversations are possible. Exposure takes place under the guidance of the therapist in the safety and privacy of the consulting room, without the patient needing to go out the door. The situation can be customised for the patient. The therapist sees what the patient sees on the exposure and can directly influence the number of virtual characters, as well as the emotions and conduct displayed. Thanks to these customised scenarios and their real-time control by the therapist, the expectation is that the quality and effectiveness of the treatment is positively influenced. The latter was also shown in a Randomized Controlled Trial (RCT) conducted by Pot-Kolder et al. (2018), Pot-Kolder (2020). They investigated the effects of virtual reality-based cognitive behavioural therapy (VR-CBT) on paranoid thoughts and social participation of patients with a psychotic disorder. The results from this study (*N* = 116) suggest that the addition of VR-CBT to standard treatment can reduce paranoid ideation and momentary anxiety in patients with a psychotic disorder. The use of VR can also contribute to improving the way people deal with stress. This is apparent from a study by Maarsingh et al. (2017). They conducted a pre-test post-test pilot study to investigate the intervention “Stressjam.” This intervention combines VR, biofeedback technology and applied games to provide a personalised digital coach to train stress regulation and to develop a new stress mindset in which stress can also be healthy. This study included 27 psychiatric patients and 55 healthy employees. Both groups improved significantly on their stress mindset after playing Stressjam.

Studies have shown that VR has added value in the treatment of people with mental complaints such as anxiety, paranoia and problems in dealing with stress. In addition to the fact that VR seems useful in the treatment of mental complaints, there is also evidence that VR can contribute to learning skills more quickly. In a study conducted by PwC, a global network for business services (PWC, 2020), a VR soft skills training was investigated. Employees took the same training in one of three learning modalities: classroom, e-learning and v-learning (VR). The participants in the VR condition became proficient four times as quickly, were 275% more confident to apply skills learned after training, 3.75 times more emotionally connected to content than the classroom learners and four times more focused than their e-learning peers. These survey-based results show that VR seems to help to upskill faster. Other studies (Araiza-Alba et al., 2021) link the learning of problem-solving skills through VR to the theory of embodied cognition (Anderson, 2003). Thanks to the VR environment, the user does not have to imagine a situation. As a result, one does not need to burden their cognitive capacity unnecessarily. This ensures that the cognitive load decreases and there is more cognitive space for solving problems.

But why does VR seem to work? A key aspect of VR for psychotherapy mentioned in Hacmun et al. (2018) is the ability to induce a feeling of “presence” in the computer-generated world experienced by the user. Kaimal et al. (2020) describe that the immersive VR environments create a space to “practise” psychological and behavioural concerns and prepare for adaptive responses in the real world without the distractions. Their findings indicate that VR-based self-expression is an embodied visual expression which offers a novel medium for art therapy. Geraets et al. (2021) described how VR enables a content that is not possible in real life. Hacmun et al. (2018) referred to this as the imaginary experience of the unreal that is made possible through VR. VR has the potential to make psychiatric treatments better using aspects such as embodiment, self-led interventions and changing perspective technology. Because people can practise, experience, and discover in the artificial world with the help of the telepresent therapist, the participants wind up in the zone of proximal development (Vygotsky, 1978). People may also be able to practise on their own if they choose, moving towards the zone of unaided learning. A more recent study of Hacmun et al. (2021) focused on the experiences of seven expert art therapists, all of whom experimented with creating visual art in VR and as observers. The results indicated that the therapists foresee substantial potential in the novel VR medium for art therapy, and highlighted further research directions needed to determine how the virtual medium can be used to treat real-world problems. VR seems to have added value, both in the treatment of mental problems and in learning processes. In most cases the therapist controls the virtual space from outside and follows the client in the virtual world while he or she undergoes treatment and practises. Here it seems to be a shortcoming that the therapist really takes part in the virtual space, and is able to apply his or her own methodologies, comparable to how this ordinarily takes place in his or her own physical therapy space. This asks for the possibility that the therapist steps into the virtual world together with the client. The virtual space must be of a freely usable nature, in which numerous intervention options are available. For arts and psychomotor therapy this means possibilities in the area of games, interaction, creativity, sound and the availability of plenty of materials to carry out these activities. Because both the therapist and the client, or the group of clients, can each step into the virtual space, it is possible to work remotely. Each of them can step into the remote therapy from his or her own location. The therapist is then actually available remotely in the therapy. We also refer to this as being telepresent, or simply telepresence.

The challenge is to provide access to this innovative value for the users, both therapists and their clients, and right now this is the bottleneck in the present applications. The chance is small that a solution is proffered by the market. For suppliers of eHealth platforms, starting from the earnings model, it is less interesting to focus on a relatively small target group of arts and psychomotor therapists. This asks for collaboration between arts and psychomotor therapists and parties working on the development of applications.

This project came about based on this context. The research questions were: (1) How can arts and psychomotor therapists become capable of acting in a virtual arts therapy space? (2) What substantive applications do they see for this virtual therapy space, what possibilities or limitations do they see and what preconditions must be met? What technical or substantive adjustments to the virtual therapy space are needed to increase deployability, so that arts and psychomotor therapists can put them to better use for the therapeutic objectives of their clients, and which the arts and psychomotor therapists can enlist better for the therapeutic goals of their clients? The goals of this project, “Telepresent as arts therapist,” were for arts and psychomotor therapists to become capable enough to act remotely and to gain more access to tools that connect better to arts therapy. For this project a subsidy was given by ZonMW on the basis of the COVID-19 programme “Science for practice.” This took place from March 2020 to March 2021.

## Materials and Methods

In this action research, individuals from various organisations are motivated to make a change or to learn to deal with new societal developments. To answer the research questions, participating arts and psychomotor therapists came together as representatives of the work field to jointly analyse the problem, develop a change and implement it in the therapy. This is known as a third-person action study (van Lieshout et al., 2017). Action research is both a design and a strategy for change. The actions of the arts and psychomotor therapists are central and are subject to change.

In this action-oriented study, use was also made of the Lean Startup Method (Ries, 2011). The heart of the Lean Startup can be learned quickly: what works and what does not. Assumptions are tested efficiently in practice on the basis of a Minimum Viable Product (MVP). The experience and knowledge that arises from this (data) are evaluated (learning) and assist in the continued development of the product (new idea which leads to new MVP). Each time this circle “build-measure-learn” is applied. This expedites the innovation and limits costs. The starting point was indeed that, using this approach, arts and psychomotor therapists would become more skilled in the use of the telepresence tool. This approach produces a cycle in developing literal application know-how (arise/emerge, internalise, automate) and procedural knowledge, knowing how to enlist the tool in your own context (Ebbens, 2004). Here, own context also refers to a therapeutic objective. The therapists could choose one of three digital tools about which they could learn more during the project: virtual reality (VR), arts therapy space, a 3D painting application and/or an emotion-regulating monitoring app. For a good overview and depth of content, we report in this article only about the virtual therapy room as a means to be used in arts therapy. The other applications will be incorporated in other publications.

### Participants

A maximum of twenty arts and psychomotor therapists working in mental health care in Netherlands could participate. They were recruited via the various professional organisations, via the placement organisations of the various arts therapy training courses, via newsletters and websites of two lectorates and via social media (LinkedIn, Twitter, and Facebook of the creative therapy course Arts Therapy). Participation was open to all arts and psychomotor therapists from the work field and to arts therapy teachers. Five of the twenty arts and psychomotor therapists, with an average age of 42.2 years (range 36–53 years, SD 6.7), chose to work with the virtual arts therapy space. These five arts and psychomotor therapists (one music therapist, man), two drama therapists (one man and one woman) and two psychomotor therapists (one man and one woman) worked in various sectors of specialised health care (*s-ggz*), focusing on rehabilitation, personality issues, and/or their own practice. The arts and psychomotor therapists have between 5 and 25 years of professional experience. The participating arts and psychomotor therapists signed an informed consent form.

### Procedure

#### Preparatory Phase

Interested individuals were selected in a preparatory phase. They went through an online process consisting of three knowledge clips and four digital questionnaires. Via this process we selected a motivated group of participants. There were 123 responses to the first form, 86 to the second, 56 to the third, and 33 to the last one. In these forms they were given an explanation of the tools and the Lean Startup Method. Using self-made questionnaires (see Supplementary Material), data were collected relating to their attitude towards digital tools and how they imagined that they would be able to use them in their therapy.

#### Sprint Sessions

Over a period of 3 months we organised three sprint sessions (once a month) according to the Lean Startup Method (Ries, 2011) and a presentation/evaluation session. In the meantime extra practice and explanatory sessions were held. A digital work environment was set up for the participants. Technical knowledge and skill were hired from companies that already had this in-house, and desired adjustments could be made immediately. The sessions could be classified as follows:

• Sprint 1: Generating the first idea to be carried out and creating the first MVP. Completing a test card, a template with which people can work out the first idea step by step on the basis of items formulated in advance.
• Sprint 2: Processing the experiences and knowledge from sprint 1 and working it into a new MVP to be tested in practice. Depending on the possibilities in their own practice, this was done with clients or with colleagues working together.
• Sprint 3: Processing the experiences and knowledge from sprint 2 and working it into a new MVP to be tested in practice.
• Presentation/evaluation session: The participants presented their experience, observations and acquired knowledge in a film or PowerPoint presentation in which they also gave their longer-term perspective (what will they definitely enlist/what requires further study?).

The arts and psychomotor therapists practised with one another or involved their clients, if possible, but only when this fit in the current therapy process. At the same time the therapist informed the client about the project and how it was set up. The arts therapist and his or her own experiences and observations relating to clients are central in this study. After the four sessions, the participating arts and psychomotor therapists were interviewed by the researchers involved.

### The Virtual Therapy Space: “VR Health Experience”

**FIGURE 1:**
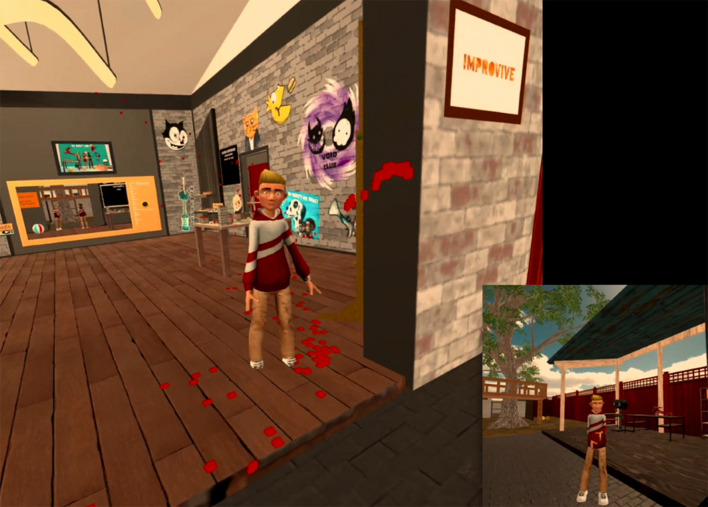
The virtual arts & psychomotor therapy space “VR Health Experience”.

The virtual arts therapy space “VR Health Experience” was designed and prepared as a basic multi-player environment, equipped for arts therapy, in collaboration with parties that concentrate on technological developments and their effects. The VR Health Experience (previously called VR Body Lab; iXperium, 2021) is a virtual environment in which you can hear and see each other and move about in the same digital space. The therapist can take part with VR glasses or can watch via the computer using a screen, and can therefore intervene in real time during the session and work experientially, as if you see one another in the therapy space (see Figure 1). This increases the sense of being present and in contact with one another. During the project, adjustments could be made and problems corrected in response to input from the sprint sessions.

### Interviews

The participants were interviewed individually about their experience with the VR Health Experience application. The semi-structured interviews were based on a topic list. The topic list consisted of a number of questions that formed a guideline for the discussion (see Supplementary Material). The topic list was aimed at the various stages in therapy (preparation, terms/conditions, design, planning and implementation, evaluation), possibilities and limitations. The topic list was used to prevent important topics from being neglected and to bring fluency to the conversation if necessary. Participants were also asked to articulate their experiences in discovering the VR therapy room and how they thought they could use this in their arts and psychomotor therapies, their experiences during the sprint sessions and how they evaluated their learning in these sessions. Each interview lasted about 45 min and each respondent was interviewed once. The interviews were recorded by audio recording and transcribed.

### Data Collection and Analysis

The online questionnaires and interviews provided the data to be analysed. Data from the questionnaires were analysed on participants’ characteristics, and averages, frequencies, and before and after measurements were compared. The interviews were subjected to a qualitative, thematic content analysis (Verhoeven, 2020) and followed the steps (1) explorative encoding, (2) relevant encoding, (3) unique encoding, and (4) provisional grouping. The data were thematised in this way. Films and other visual material were collected to be able to transfer results to each other and to third parties, such as symposia for colleagues and interested parties.

The first research question, “How can arts and psychomotor therapists become capable of acting in working with the VR therapy space?” was addressed by evaluating experiences and analysing the scores during and after the training. The second research question “How can arts and psychomotor therapists work with this application in their therapy for the therapeutic objectives of the client?” will be addressed in the qualitative thematic analysis of the interviews held with the participants. This included substantive applications, possibilities/limitations and preconditions. Lastly, two case vignettes of patients’ experiences in group therapy using the VR therapy room, as observed by one of the therapists, will be described to illustrate the use of the VR Health Experience in the clinical practice of the participants.

## Results

### Becoming Fully Competent

One aspect of the action study was exploring whether the working method which had been developed for the first time would comply with this objective. Participating arts and psychomotor therapists indicated how the project contributed to their degree of competence in working with the tool. The results are shown in Table 1.

**TABLE 1:** The degree of competence of the arts and psychomotor therapists before and after the project (Mean/SD/Range).

Item	Before project		After project	
	M(SD)	Range^*a*^	M(SD)	Range^*a*^
How competent do you feel you are in the use of online tools?	3.4^*b*^ (1.1)	2–5	4.8^*c*^ (0.4)	4–5

*^a^Range 0–6: 0 = “not at all competent,” 6 = “highly competent”. ^b^3.4 = between “insufficiently competent” and “somewhat competent”. ^c^4.8 = between “somewhat competent” and “fairly competent”.*

At the end of the project all participants were asked to rate a number of statements relating to the use of digital means and giving online arts therapy, using a 5-point answer scale (1 = “*disagree completely*” to 5 = “*agree completely*”). The arts and psychomotor therapists gave the highest score to learning new skills; “I have learned new skills since I offer online arts therapy”: 4.4 (SD.55; range 4–5). A 4.4 (SD.55; range 4–5) also went to the statement “it is possible to act and to experience in an online environment.” The statement “The treatment techniques I am familiar with are easy to apply via online arts therapy” had a score of 4.2 (SD.84; range 3–5). To the statement “I am positive about giving online arts therapy” the score is: 4.0 (SD.71; range 3–5). A statement with the same score said “I have an affinity with digital technology” 4.0 (SD.71; range 3–5). The final statement stated “I experience higher work pressure when I have to offer online arts therapy,” with a 3.0 (SD.55; range 2–4).

### Substantive Applications and Preconditions

#### Conditional Basic Knowledge

A number of participating arts and psychomotor therapists noted that it is not always necessary to be fully aware as a therapist of how the VR glasses and the application work in order to enlist this in arts therapy. A statement, for example:

“In part, it is nice to know in advance how it works when you set to work, but somehow it is also interesting to research together how it works, and then the theme of frustration tolerance may come up” (participant 1, drama therapist, 44 years).

They said that the therapist himself or herself must be capable of launching the VR glasses and making them ready to use to work with the application. A space of two by two metres is already enough. For this, the client must know how to move about in the VR space, often referred to as transporting or teleporting. The participating arts and psychomotor therapists said that clients proved to be reasonably skilled digitally. This made it possible to start individual sessions fairly quickly. At times when a client was less skilful, in an individual session a closer look could be taken from an arts therapy framework. Certain therapeutic themes that came forward could be transposed into a new learning situation for the client by means of an intervention.

#### Material

Participating arts and psychomotor therapists said that the avatar (representation of the person in the virtual world) is partially a given where the facial expression plays less of a role than normal in a face-to-face session. One of them (participant 4, psychomotor therapist, 39) said:

“The facial expression of the avatar was already created, and so greater attention went to its body posture” (participant 4, psychomotor therapist, 39 years).

The majority of the participating arts and psychomotor therapists said that the app works in an intuitive and user-friendly manner and is a good and attractive alternative for video calls and online therapy. At least two pairs of glasses were deemed to be essential. If more glasses are not available, then watching on a screen on which the therapist sees what the client sees in the glasses is a good alternative. Watching together with the client is seen here as a necessary condition for conducting the therapy in this manner.

#### Advantages of the VR Therapy Space

Firstly, in the VR space, people can come within the one-and-a-half metre distance of each other (relevant in this day and age of social distancing). Another advantage of the VR space is that it is possible to enlarge or reduce the avatar. This can be used to evoke body signals or to adopt different positions in the interaction. For example, it can be connected to different modes in schema therapy: one person in a looming, punitive parent mode, the other in a small, vulnerable child mode. It was also perceived as useful that everything in the VR space can be turned upside down. At that point people certainly want to see the entire space clean and tidy again by pressing a single button. Clients said that they thought it felt quite odd that they could stand outside of the space, as it were. This endless light blue environment where people wind up looked like heaven, according to one client. He perceived it as very serene and calm. Another client experienced a sense of floating or hovering when he leapt from the tower to the ground. These experiences are unique to working in the VR space. They cannot be evoked in the “real” world, or are at least very difficult to summon.

#### Which Approach Is Indicated

The participating arts and psychomotor therapists see indications for target groups that suffer from depression, anxiety, psychosocial, and/or identity problems. According to the participating arts and psychomotor therapists, VR Health Experience can definitely be enlisted transdiagnostically. It might also work at a low threshold, for example for clients who have a difficult time coming to the therapy location on account of a physical limitation and/or have great difficulty coming to the location for all manner of reasons; this would allow them to come to therapy after all.

“This VR therapy space would be very nice for people who are less mobile, or who have reduced mobility. It allows them to perceive that they are moving and thanks to this, they feel and experience greater freedom” (participant 3, music therapist, 53 years).

Group therapy is seen as a possibility. However, it must be taken into account that it is more difficult to organise and that not all clients can enter the group at the same time (for technical reasons). If all clients step into the app at the same time, the software cannot handle the processing capacity. Then the application may get stuck. As far as an indication goes, it is important to point out that first and foremost, the VR therapy space serves the objects of the client; after all, the VR therapy space is a means, not an end in itself.

The majority of the participants expressed some doubt as to the use of the of VR Health Experience with clients who are prone to psychoses, clients who are mentally handicapped, schizophrenic or very young, perhaps around four or so. These doubts are based on what they thought and not on their own experiences thus far. There are other doubts on the part of the participating arts and psychomotor therapists as to indications or contraindications for clients with a gaming addiction, and to the target group of geriatric clients.

#### Working Methods

Frequently used themes are recognising body signals, pointing out/defining limits, distance and closeness, strengthening self-respect, guided fantasy, role playing, psychodrama, working together, a space of one’s own, experiencing play activities, and pleasure. A working method such as a controlled approach, improvisation, and indicating limits can be carried out very well in the digital world.

“In fact I think it’s suited to all movement and play-focused working methods, if you do your interventions well. I wouldn’t be able to think of anything for which I couldn’t use it” (participant 2, psychomotor therapist, 39 years).

The participants do not think that this application is equally suitable at present for all art therapy disciplines. People are the most enthusiastic about psychomotor therapy and drama therapy. The VR Health Experience was initially developed for psychomotor therapy, which can be seen in the activities present: building, sports and play activities. Being able to “dress up” as another avatar and the possibility of role playing fit well with drama therapy. Arts therapy is also considered a possibility, although it is advised that more possibilities for drawing, painting or spatial visual work should be created. Possibilities are seen for dance therapy, although controllers for the legs would be considered useful. For music therapy, there could be possibilities if a more specific musical offer was added in the VR therapy space.

#### Difference With Face-to-Face Therapy

The participant therapists do not view this as a replacement for face-to-face arts therapy, but as variety, as supplementary. The contact was perceived by most arts and psychomotor therapists as direct, also when there was only one pair of glasses present for the client. One of the arts and psychomotor therapists commented that he would even start working more directively and confrontationally in the VR environment. Clients also seemed to be triggered differently, as is apparent from the following quote:

“What I thought was very unique was that I saw an entirely different client. I saw him rally, he was trying to learn through play” (participant 2, psychomotor therapist, 39 years).

So although there definitely is contact, it is also definitely different contact to a face-to-face situation. The participants said that people need to ask more often if what they think is correct, or think they are observing, because people noticed that more is non-verbally visible in face-to-face therapy in the physical arts therapy space.

#### Can the VR Therapy Space Be Used in Arts Therapy?

Everyone wholeheartedly agreed that this app might be a new tool for arts therapy. The arts and psychomotor therapists indicated that people can work online with techniques that are less well possible in physical space. It is their experience that in this online manner, a play reality is created that makes a huge appeal.

“This is a cool type of therapy (…). You know it’s not real, but even so, there is contact and people create something together” (participant 2, psychomotor therapist, 39 years).

Clients can feel safer in VR therapy space, as is apparent from the initial experiences. One of the therapists commented that arts and psychomotor therapists can distinguish themselves by the experiential manner of working when using VR. This is an added value of arts therapy in VR compared to a number of other disciplines that work with VR, for example. The participants also indicated that more connection is felt in working in the VR therapy space compared to working with video calls.

“You feel other things while working online and at a distance, different to arts therapy physically, on location; you deal with your body in a different way” (participant 5, drama therapist, 36 years).

#### Expected Effects

Based on experiences thus far, the VR experience has proved to have a strong impact on the person who undergoes them. For example, it diverts your attention from day-to-day stress because you are briefly in a fantasy world. This “brief escape from reality,” many clients like it a lot: a moment of relaxation and discharge, after which they feel recharged. Participants see behavioural change in the client in the online world, a connection is felt, clients recognise body signals and you can work on a range of treatment goals. Participants think that this manner of working may have effect in working on observation and treatment goals such as: recognising body signals; scouting, exploring and asking yourself about feelings; learning to express and to regulate emotions; recognising boundaries; regulating aggression and stress; increased assertiveness; learning pleasure and enjoyment; exposure in the context of trauma treatment; and improving cooperation/collaboration skills. Participants also see possibilities to implement arts therapy in combination with schema in the VR therapy space and to work on experientially recognising various modes.

#### Useful Technical Adjustments

Possible additions to the VR therapy space/VR Health Experience to increase usability for the therapeutic goals of clients, according to the arts and psychomotor therapists:

• able to play your own music playlist;
• able to be in the separate spaces, individually or in smaller groups, and perhaps discuss the follow-up;
• name plates next to avatars during a group session;
• able to type something rather than write it on the wall;
• putting on different clothes (the female avatars might be considered too distracting or sexy). When people change clothes, a clearer role can be chosen (think of archetypes) or the client can look more like himself or herself;
• able to sit down/chairs in the VR space;
• able to use your legs, so that you can kick a ball around.

To allow the VR Health Experience to connect well to all arts therapy disciplines, it is necessary to add options such as visual and musical possibilities, sculpting, more painting and drawing materials, and musical instruments. For dance therapy, the possibility of using your legs ought to be more sophisticated.

### Cases

The following case vignettes describe the experiences of two clients from the observational point of view of the therapists. These clients have complaints in the area of self-image, emotion regulation and contact with others. Because of the corona pandemic, the participants had a blended-care treatment, both online and face-to-face on location.

#### William’s Case

William (45) has individual psychomotor therapy for anxiety complaints. He has difficulty in making contact with his body. How he copes: he has a strong avoidance urge. In psychomotor therapy spaces, he is friendly and polite. He laughs a lot, but experiences very little in his body. He checks a lot of things that I - the therapist - want him to do, and he spends a lot of time in his head. Together we explore the VR-Health Experience space and together, we throw a ball back and forth on the “sports field.” The ball lands in the goal and he wants to get it out, but each time he lifts the goal instead of the ball. I hear grumbling and see William pick up the goal and put it back, pick it up and put it back. His movements become busier and bigger. I stop William when I see his very obvious body signals. He recognises muscle tension, pressure on his chest, his forehead is tense and he has something in his stomach. Together we point out the body signals, using colours on the available body silhouette, so that he can see from a distance what he feels in his body. Working with several different colours, we were able to make a distinction in body signals belonging with various emotions. William is surprised that he has observed so much. When he stood at the top of the tower, he could feel anxiousness in his legs and chest (red). Pleasure and delight when he plays with the ball - this is also in his stomach, but a bit higher, and in his chest (yellow). And then the irritation when he wants to pick something up and is unsuccessful, in the tense muscles in his shoulders, arms and hands (blue). He would like to take home a photo of this. The next time, he says he caught himself often asking what it is he feels in his body. At home, he practised twice on his own in VR-Health Experience to observe body signals. The next time the session is face-to-face. We both see that it is harder for him to observe the body signals. I notice that he is more focused on contact with me. He says that when he wears the VR glasses he has fewer stimuli, less distraction and more focus.

#### Sasha’s Case

Sasha (34) takes part in a schema therapy group together with seven other clients. She has an avoidant personality disorder. Sasha has a difficult time coming in contact with others; because of her past, she feels a great deal of mistrust. Her coping strategy: when she feels ignored, she gets angry at others.

On the basis of experiential exercises, they learn how to gain insight into patterns, how these are expressed in modes and how they can handle this. The project described in this article offered the therapist working here an extra possibility: borrow several sets of VR glasses in order to work with a group in VR-Health Experience. These sessions were entirely online; clients were at home, the group started out with video calls and then switched to working with the VR glasses. The past sessions with video calls were difficult for the entire group, including Sasha. You can see one another, but it is not at all clear whether a person is really looking at you. This brings Sasha more into her avoidance mode, where it is more difficult for her to make contact. She indicates that things are going pretty well with her. In the VR space she chooses an avatar that seems to suit her personality: one with the most “inconspicuous appearance.” We start out by making a circle, the assignment being to hold each others’ hands and to look closely to the left and the right. Both the group and I myself and my colleague think this is a unique experience. Everyone is 20 kilometres apart, but even so, here we are, standing together hand in hand. I hear the group chuckle and make cheerful sounds. I hear Sasha shout: “It’s strange! You standing here, it almost feels real!.” Other group members agree. They feel more connection. Wonderful to see how Sasha has become part of the group in this situation and context. Then I hear, “Sasha, can I give you a hug?” And it’s okay. The group experiments wildly: making connections, touching one another. Because the sessions largely took place online, the group had not yet been in contact with each other in this physical manner. We devoted the rest of the session to the happy child. We discovered the space together and played games like 1-2-3 piano, tag, ball games and hide and seek.

When playing hide and seek, Sasha hides in the high tower. A group member is “it” and finds the other group members one by one. People are talking all at once, giving hints, including the group members who are still hiding. In VR-Health Experience it makes no difference, because you can’t hear where the sound comes from. So you can’t reveal yourself or your hiding place. Sasha says nothing at all, she just waits. The person who is “it” thinks she has found everyone and asks them all. Because there are so many avatars, it is difficult to see who belongs with which of them. Now another person is “it.” This person thinks she has found everyone and asks around. Sasha is silent. Because there are many avatars, it is difficult to see who goes with which avatar. A new person is appointed “it”. A little later Sasha comes forward and says that she wasn’t found. In this situation Sasha was overlooked, and this happens fairly often to her. When I talked about this with Sasha, it turned out that a mode was active in which she was “testing” other people and found confirmation of the fact that she had not been seen. This was an observation we had not made earlier in the group. Together with Sasha, we looked at how this happens in other situations and how, in all these situations, she can make her needs clearer and can start building up her contact with others.

## Discussion

With respect to the first question of how arts and psychomotor therapists can become capable of action in working with a virtual arts therapy space, it can be said that the method and training used clearly contributed to this. The participating arts and psychomotor therapists learned that they had acquired new skills and that they had started to feel more capable by taking part in this project. They saw possibilities to apply arts therapy in this way, to use telepresence, and people were positive about it. Broadly speaking, the online training seems to be worth repeating.

The second question concerned what substantive applications arts and psychomotor therapists see for the VR therapy space, with attention to possibilities, limitations and preconditions. The following conclusion can be drawn. The participants found that, to use the HR Health Experience in arts therapy, basic knowledge of the tool was sufficient; investigating and discovering together with the client(s) also proved to be valuable. The presence of several sets of VR glasses was found to be important in making optimal use of the tool. The most important advantages of VR Health Experience were named as follows:

• being close together even though in reality people were at a distance from each other (and could approach one another very closely in a time of social distancing),
• assuming a role via an avatar they had chosen,
• being able to make their avatars larger or smaller, which offered stimulating therapeutic possibilities,
• being able to adopt several perspectives, and
• being able to seek out experiences that cannot be carried out in the normal world or are not wise (jumping from heights, a sense of hovering/gliding, floating, making a mess).

There seem to be indications for the use of the VR Health Experience for a wide range of target groups and for both individual and group therapy. This would seem to be indicated particularly for people who are less mobile, or not at all, and people who have a hard time getting to therapy. People have a number of assumptions about contra-indications for clients who are extremely sensitive to stimuli, who have a gaming addiction or an intellectual disability, for people who are very young or very old and who may have physical or mental difficulties in using them. Whether these assumptions are correct remains to be seen and further research needs to be done in this regard.

As to work formats, at present, VR Health Experience is mainly adjusted to movement and game/play-oriented work methods. Role playing is possible, as is drawing, building, etc. This application is therefore now best suited to drama and psychomotor therapy and less suited to art therapy, dance and music therapy. An important disadvantage is that it still needs some technical adjustments and substantive supplements to make it equally suited to all art therapeutic disciplines. The other disciplines can be used in the VR environment, but the environment itself needs to be further developed with more possibilities such as musical possibilities, sculpting, painting and drawing options and the possibility of using your legs for dance movements.

The participants learned that the added value of VR Health Experience consisted of a new trigger for clients in which they seemed to start playing and experimenting more. Non-verbal contact - mimicry, for instance - is more difficult in the VR world. On this account both the therapist and the client were expected to communicate more clearly (being more direct, more confrontational). People see that the VR space can be used well for arts therapy, but that some things are possible that are not possible, or are not really possible in an ordinary therapy space. The play reality thus created makes a great appeal to the person who is in it and increases the space in which to play. Some clients seemed to feel safer in the VR space than in ordinary therapy space. Generally speaking, people felt connected with each other, recognised body signals and could also work at a distance on a number of treatment goals via telepresence.

The outcomes of this study impact on clients who have arts therapy. Certainly in times in which therapy on location is not possible, the mental health and/or welfare of clients can be positively influenced by offering arts therapy at a distance and working with digital means such as VR. Experiences show that working with VR is a powerful therapeutic means: clients often rapidly become enthusiastic and are open to it. The impact of this project on participating arts and psychomotor therapists is that in practice, they tend to start working with VR tools sooner, depending on the local possibilities.

For a number of years, health care insurers and healthcare facilities have attached importance to the development of eHealth interventions. This means there is a real chance that after this project, there will be opportunities for further development, research and implementation, including after the measures to combat the pandemic. However, obstacles came forward in practice during this project. The arts and psychomotor therapists observe that they are in a work field where innovation is difficult. The time and the room they get for financial investment is very limited, and innovations do require extra time and financial means. The organisations also have some hesitation about being able to maintain privacy rules in working online. A number of organisations thought that trying out VR would be too much of a burden on clients. Obviously, care and consideration are needed in all fields. Even so, it seems that the unknown aspect of enlisting VR in therapy can also have an inhibiting effect. In the digital time we live in, working with VR glasses could also be regarded as the umpteenth therapeutic means, along with numerous other techniques and work forms enlisted, which clearly need to be well researched. The economic motive often named (Geraets et al., 2021): in the long run, the use of VR can ensure that the costs for mental healthcare can be limited, may well be doubtful.

This study clearly had a number of limitations. Firstly, the number of participants was limited and the duration of the project was fairly short for both learning to work with the tools and applying them properly in therapy. Both points argue in favour of a follow-up study with more therapists and a longer period in order to arrive at more enrichment and development. Even so, with this study we took a first step and based on the initial results, we can show what is in principle possible in arts therapy using the VR Health Experience. Secondly, despite the fact that a great deal is already possible in this application, the VR Health Experience is also a sort of prototype. This asks for adjustment and development to ensure an optimal connection to all forms of arts therapy and for this, the professionals are also needed to indicate their wishes and requirements. A third limitation is that the participating arts and psychomotor therapists may take a more positive stance towards working online than the larger group of arts and psychomotor therapists in the work field. After all, they were the ones who decided to take part. However, we can regard these participating therapists as the “early adopters” (Rogers, 2003): they clear the way for other arts and psychomotor therapists and perhaps for professionals in other disciplines as well, to develop themselves in this field.

Strengths in this study are its innovative character and the fact that VR Health Experience was also tried out with clients. As far as we know, this was one of the first studies in which therapist and client were truly in the virtual space at the same time. Usually the client is in the virtual space and the therapist steers the process remotely. Being together in the virtual space in a therapeutic situation is pioneering and offers unusual possibilities, in our estimation. The study of Geraets et al. (2021) reviews current advances in immersive VR-based therapies for mental disorders. They state that VR is a potentially revolutionary tool for psychological interventions. It has the potential to make psychiatric treatments better and more cost-effective and to make them available to a larger group of patients. However, Geraets et al. (2021) also state that this may require a new generation of VR therapeutic techniques that use the full potential of VR, such as embodiment, and self-led interventions. VR Health Experience makes use of this embodiment and makes it possible for clients to practice by themselves. Transferability of the present study is strengthened by the fact that it was conducted among experienced arts and psychomotor therapists from different mental health care institutes. A strength of the online training for the project was that each participant could join in at his or her own level. The Lean Startup Method fit in well with personal wishes. It provided a sound basis and made the steps tangible and clear.

VR-based interventions are promising, but further research is essential. Well-designed studies are needed that investigate efficacy, efficiency, and cost-effectiveness of VR interventions compared with current treatments (Geraets et al., 2021). This will be crucial for implementation and dissemination of VR in regular clinical practice. A recent study of Bedir and Erhan (2021) on the effect of a VR training programme on the performance of athletes showed that the participants in the VR condition improved significantly compared to those in the video training condition. However, this must be studied more closely before making any statements about it. The present study on the use of VR in arts and psychomotor therapies presents promising signals. For further research we recommend more participants, therapists and clients, over a longer period of time. As we go along, the content and technique of VR Health Experience has to be adjusted on the basis of instructions of the arts and psychomotor therapists of possibilities in content and technique. We would like to develop VR Health Experience so that it will become applicable equally to the various arts and psychomotor therapies forms. After development of the VR Health Experience, we advise further intervention development of the VR application of existing and new arts and psychomotor therapies interventions. The effect can then be studied on the basis of sound descriptions of interventions.

Looking at research developments, the integration of VR and (neuro) physiological measures could be helpful. VR combined with physiological measures, such as heart rate variability (HRV), which measures the stress level as neuro-feedback, might lead to more insight into sensorimotor areas, core emotional centres, and reward-related centres in relation to arts and psychomotor therapy (Di Dio and Gallese, 2009; King and Parada, 2021). Mobile brain and/or body imaging during therapy (MoBI, King and Parada, 2021) could allow for more direct study of the neurophysiological signals associated with behaviour in psychotherapeutic encounters, to study *interoception in the wild* and the effectiveness of the arts and psychomotor therapy interventions. MoBI takes full account of the body and environment in which cognition arises. An interesting aspect, as most neuroaesthetics studies do not examine the ecological setting of the therapy setting in which encounters and the dynamic change are most important. Arts and psychomotor therapies research is and probably remains challenging because multiple psychological processes are in involved in therapy and is even made more complex by the additional components of artistic expression and body movement. However, the combination of VR therapy and (neuro) physiological measures could move the field towards more insights, more scientific validation and to the further implementation of successful interventions.

In conclusion, the participants in this project experienced the innovative value of working with VR in arts and psychomotor therapies by means of the VR Health Experience. They regarded it as an extra possibility for arts and psychomotor therapies and also being able to continue this at a distance. Both the arts and psychomotor therapists and their clients felt the impact of working in VR. The virtual world was often perceived as being safe. This artificial as-if world challenged many arts and psychomotor therapists to experiment with new behaviour. It offered them a powerful playing field on which to practise and the threshold to do so became lower, precisely because it is in an artificial world where other things are possible. For arts and psychomotor therapists, it became clear that they can give therapy at a distance in this way, where their experience is still that people are close together. Telepresent; at a distance but very much present.

## Data Availability Statement

The raw data supporting the conclusions of this article will be made available by the authors, without undue reservation.

## Ethics Statement

Ethical review and approval was not required for the study on human participants in accordance with the local legislation and institutional requirements. The patients/participants provided their written informed consent to participate in this study.

## Author Contributions

SH and JK are responsible for conception and design of the study. JK lead the execution of the sprint sessions. NJ was the research assistant. SH supervised. NJ organized the data. SH and NJ wrote the manuscript. MG provided cases and participated in the study. All authors contributed to manuscript revision, read, and approved the submitted version.

## Conflict of Interest

The authors declare that the research was conducted in the absence of any commercial or financial relationships that could be construed as a potential conflict of interest.

## Publisher’s Note

All claims expressed in this article are solely those of the authors and do not necessarily represent those of their affiliated organizations, or those of the publisher, the editors and the reviewers. Any product that may be evaluated in this article, or claim that may be made by its manufacturer, is not guaranteed or endorsed by the publisher.

## Footnotes

1 The Dutch Association of Arts Therapies and Psychomotricity (FVB, 2021) comprises of six professions: art, drama, dance, music, play therapy, and psychomotricity. Psychomotor therapy is a distinct profession in Netherlands with its own education programme and its own professional association (Nederlandse Vereniging voor Psychomotorische Therapie (NVPMT), 2021).
