# Supplementary material for: VR Health Experience: A Virtual Space for Arts and Psychomotor Therapy
Source: Front Psychol. 2021 Sep 14;12:704613. doi: 10.3389/fpsyg.2021.704613 (PMC8476779; doi:10.3389/fpsyg.2021.704613)
Supplement: Supplementary file 1 [file Table_1.DOCX]

**Interview topics VR Health Experience**

Preparation

- Do you need technical skills as a therapist (maybe you want to follow a course before you start?)
- How many preparation time did you need before you could start with VR therapy?
- How was it to charge the VR glasses and to replace the batteries for the controllers?
- How many sessions to get knowledge about VR Health Experience? or can you practise an exercise without any experience
- What kind of physical space is needed?

Material

- How is it to wear glasses while wearing the VR glasses?
- What is your opinion about an extra VR glasses for you as a therapist?
- Do you want an extra screen to watch you patient while you are not preparing? If yes/no, why?
- Is there any other material that might be useful in a session?

Indication setting

- What kind of patient group(s) do you recommend this application
- Do you have some contra-indications
- Is this application for individual sessions, or can you use it in groups?

Therapy activities

- In what therapeutically way could you use the VR Health Experience?
- For what kind of arts and psychomotor therapies is this application available for?

Therapeutic attitude

- How did you experience the contact between the patient and the therapist?
- Is there a difference in therapeutic attitude comparing a live session and a VR Health experience session?

Use in arts and psychomotor therapies

- Do you think VR Health experience might be a new tool in arts and psychomotor therapy?
- Which opportunities/disadvantages are there for the therapist?
- Which opportunities/disadvantages are there for the patients?
- Which therapeutic goal can be worked?
- What appears to be the effects?

**Questionnaire 1. Start training**

1. Sense of competence in the field of online tools
   1. Do you feel competence by using online tools

Not at all | Hardly | Inadequate | A little | Reasonable | Excellent

1. Do you use in therapy online tools?

Yes / no

- IF YES

1. If yes, what kind of tools do you use?

- IF NO

If no, why don’t you use online tools?

1. Vision for the future
   1. How often do you see yourself using online tools in the future?

Never | Hardly | Rarely | Often | A lot

1. For which therapeutic goals would you especially like to use online tools and from what point of view? (name some options)
2. Please give your name, so we can link your answers.

**Questionnaire 2. After first training**

Please fill in the test card:

| Test card |  |  |  |
| --- | --- | --- | --- |
| Test name |  | Final date |  |
| Name practitioner |  | Duration of the test |  |
| We believe that |  | | |
| To check it, we’re going to do the following |  | | |
| We measure |  | | |
| We are right when |  | | |

**Questionnaire 3. Before session 3**

*In preparation for the 3rd Lean Start up session, we came up with an assignment in which we ask you to share what you have discovered so far. Please all complete the survey (then we will have data to analyse again).*

1. Name
2. Last name
3. What was your blind assumption?
4. Which preparation did you do in response to your blind assumption?

Tell us [short] which steps you’ve taken in preparation to test your blind assumption

1. Which requirements did you need from your preparations and blind assumption?

Mention the conditions you think were necessary to validate your blind assumption. For example: Tools, knowledge, organization, methodology

1. How did you worked on your blind assumption? Tell us [short] what you did (work phase).
2. What was your output from you work phase? And what was the effect?
3. Did you take obstacles?
4. What knowledge have you gained?

Tell us [short] what knowledge you gained or eventually found out.

1. What is your next step?
2. What do you think is needed to take the next step. [when you let go of the impossibilities].
3. What conditions are needed to be able to take the next step?
4. Make a summary of the answers you described above in a maximum of 10 lines.

**Questionnaire 4. After session 4.**

1. Name
2. Last name
3. Male/female
4. Age
5. I am: psychomotor (child) therapist | art therapist | music therapist | drama therapist | Dance therapist | Play therapist
6. Years of work experience
7. I work with the following target audience
8. Please indicate below which applies [Totally disagree | Disagree | Neutral | Agree | Totally agree]
9. I am positive about giving online arts and psychomotor therapy
10. I feel sufficiently competent to give online arts and psychomotor therapy
11. I have an affinity with digital technology
12. Doing and experiencing is possible in an online environment
13. The treatment techniques I am familiar with can be applied well through online arts and psychomotor therapy
14. I experience a higher workload when I do offer online arts and psychomotor therapy
15. I have learned new skills since I offer online arts and psychomotor therapy
16. Which telepresence form do you already know?
    1. Mail/Chat | calling | video calling | Telerobot | Gaming | Virtual Reality
17. How competent do you feel yourself in using online tools?
    1. Not at all | Hardly inadequate | A little | Reasonable | Very
18. Do you use online tools in therapy?
    1. Yes, because
    2. No, because
